# Comparison of pain relief and limb function improvement after extracorporeal shock wave therapy and thermomagnetic therapy in the treatment of low back pain

**DOI:** 10.12669/pjms.39.1.6668

**Published:** 2023

**Authors:** Tao Wu, Dun Wang, Xia Zhang, Junmei Li, Bo Yuan

**Affiliations:** 1 Tao Wu, Department of Rehabilitation Medical Center, West China Hospital of Sichuan University, 37 Guoxue Lane, Chengdu 610041, Sichuan Province, P.R. China; 2 Dun Wang, Department of Rehabilitation Medical Center, West China Hospital of Sichuan University, 37 Guoxue Lane, Chengdu 610041, Sichuan Province, P.R. China; 3 Xia Zhang, Department of Rehabilitation Medical Center, West China Hospital of Sichuan University, 37 Guoxue Lane, Chengdu 610041, Sichuan Province, P.R. China; 4 Junmei Li, Department of Special International Medical Center, West China Hospital of Sichuan University, 37 Guoxue Lane, Chengdu 610041, Sichuan Province, P.R. China; 5 Bo Yuan, Department of Special International Medical Center, West China Hospital of Sichuan University, 37 Guoxue Lane, Chengdu 610041, Sichuan Province, P.R. China

**Keywords:** ESWT, Extracorporeal shockwave therapy, Low back pain, Thermomagnetic therapy, TMT

## Abstract

**Objective:**

To compare the rehabilitation effects of extracorporeal shock wave therapy (ESWT) and thermomagnetic therapy (TMT) in patients with low back pain (LBP).

**Methods:**

As a single-centre retrospective observational study, clinical data of patients with LBP who received rehabilitation treatment in our hospital from January 2020 to May 2021 were retrospectively collected. Based on the treatment mode, the patients were retrospectively divided into two groups: the control group (patients received core muscle training + TMT, n=51) and the observation group (patients received core muscle training + ESWT, n=56). The general data of the patients were collected and the groups were matched for age, gender and pain duration. The visual analogue scale (VAS) score of pain, improvement of limb function, β-endorphin (β-EP), prostaglandin E2 (PGE2) and nitric oxide (NO) were compared between the two groups before and after treatment.

**Results:**

The VAS scores of the observation group were lower than those of the control group at one, two weeks and one month after the treatment (*P*<0.05). After the treatment, the proportion of mild limb dysfunction in the observation group was 57.14% (32/56), which was higher than 35.29% (1 /51) in the control group. The proportion of patients with severe and obvious disorders was 0 and 5.36% (3/56), respectively, which was lower than 11.76% (6/51) and 5.88% (3/51) in the control group (*P*<0.05). After the treatment, levels of NO and PGE2 in the observation group were lower, and the level of β-EP was significantly lower than in the control group (*P*<0.05).

**Conclusions:**

A combination of core muscle training and ESWT can effectively improve the analgesic effect of the treatment and promote greater improvement of limb function in patients with LBP.

## INTRODUCTION

Musculoskeletal pain refers to the pain that occurs in bones, muscles, tendons, joints, etc., and may be caused by a variety of neurological, orthopedic and other conditions.^1^ Among them, low back pain (LBP) is considered the leading cause of limited mobility worldwide.^2^ It is reported that the global point prevalence of LBP is 9.4% and is increasing with age.^3^ The costs associated with LBP vary from country to country, and are estimated to be as high as 1-3% of the gross domestic product^4^-^6^, which places an enormous economic burden on individuals and the society. Thus, LBP is a significant global health issue.

In China, core muscle training, thermomagnetic therapy (TMT) and extracorporeal shockwave therapy (ESWT) are also commonly used to alleviate the degree of low back pain and improve the effect of functional recovery. However, these different therapies have different curative effects.^7^ Recent studies showed that core muscle group training cannot only effectively strengthen patient’s core muscles, but also improve balance, strength and coordination, stabilize the lumbar spine region, alleviate the degree of musculoskeletal pain and effectively reduce the frequency of pain recurrence.^8^,^9^ ESWT has also been demonstrated to be an effective intervention for the treatment of patients with low back pain.^10^,^11^ The combination of targeted core muscle training and shock wave therapy can further improve the overall curative effect.^12^,^13^ ESWT is a non-invasive/ minimally invasive treatment which does not cause unnecessary damage to the patients during the treatment. It is a simple and convenient procedure with high safety and remarkable curative effect.^14^ However, recent studies on the effect of ESWT combined with exercise have conflicting results on whether ESWT with exercise can effectively improve patients’ functional state.^15^-^17^ Therefore, the current study analyzes and compares the effects of extracorporeal shock wave and thermomagnetic therapy on pain relief and limb function improvement in the treatment of LBP, to provide more valuable reference for the clinical treatment of this disease.

## METHODS

In this single-center retrospective observational study, medical records of 107 patients (57 males and 50 females) with musculoskeletal pain, treated in our hospital from January 2020 to May 2021, were collected. Since this period was the time during the COVID-19 pandemic, some precautions were taken during this special time: 1) The air and the floor of the hospital were thoroughly disinfected four times a day and the doors and windows wee opened for at least 30 minutes every day to maintain air circulation; 2) The operating instruments were wiped by disinfectant with an effective chlorine concentration of 2000 mg/L; 3) Patients had to wear a protective mask and undergo a temperature test when visiting a doctor; 4) After each treatment, the places of shockwave and thermomagnetic instruments contact with the patient were wiped twice with 75% alcohol, and the shockwave and thermomagnetic instruments were also wiped twice with 75% alcohol between treatments.

Age of the patients ranged from 30 years to 69 years, and the duration of the disease was two to 27 months. Patients were retrospectively divided into two groups based on the mode of treatment: 51 patients that received core muscle training + TMT comprised the control group, and 56 patients that received core muscle training + ESWT comprised the observation group. The groups were matched for general data on age, gender, and pain duration. There were no significant differences in general data between the two groups (P > 0.05), which suggests comparability. We conducted this study in accordance with the Strengthening the Reporting of Observational Studies in Epidemiology (STROBE) reporting guideline.^18^

### Inclusion criteria:

- Patients aged 30~70, regardless of gender;
- Patients with nonspecific LBP diagnosed by clinical X-ray examination and MRI examination;^19^
- No contraindications of ESWT or TMT
- Clear consciousness and stable vital signs;
- Normal cognitive function
- Complete medical records.

### Exclusion criteria:

- Patients with spondylolisthesis, spondylolysis, severe osteoporosis, lumbar vertebrate fractures;
- Patients who had prior spine surgery or receiving anti-inflammatory drugs or analgesics.
- Local skin infection or damage;
- Accompanied by mental diseases, cancer, cardiovascular and cerebrovascular diseases and other serious disorders;
- Heart, liver, kidney and other organ dysfunction.

### Ethical Approval:

All processes of this study fully comply with the relevant rules and regulations of the medical ethics committee of West China Hospital of Sichuan University (Approval number: 2021454; Date: June 22, 2021).

Core muscle group training was carried out as follows. Before the training, the weak chain test was performed to observe the weak links of the deep stabilizing muscles of the lumbar spine, and then the training plan was formulated. Patients first underwent bilateral training and unilateral intensive training in supine position, and then basic movement training in different positions such as prone position training. The training combined low load isometric contraction, dynamic and static closed chain, and the exercises were repeated 10 times/group. During the training, the patients were instructed to maintain even breathing. Patients were instructed to perform intensive exercises of gluteus medius, multifidus, internal and external oblique muscles, transverse abdominal muscles, and other muscle groups, once a day, 30 minutes per time. Patients continued to exercise for five days a week for four weeks as a course of treatment, a total of two courses.

The interventions were conducted by trained physicians who were blinded to the information about grouping. For TMT, finger pressure method was first used to identify pain points of patients, and the pain points were then marked. The operation method was as follows^20^,^21^: the pain range was defined according to the patient’s oral statement. The therapist used his fingers to apply moderate pressure to the area with strong pain, and the strength was to the extent that the patient could tolerate it. Specific pain points were determined according to the patient’s pain feeling, and thermomagnetic therapy was carried out by HOTMAGNET HM-2SC-A thermomagnetic instrument. The power on position was selected, the temperature was set to 40°C, and the treatment was performed once a day, 15 minutes/treatment. After five days of the treatment, patients were allowed to rest for two days before starting the next cycle. Patients received a total of four thermomagnetic therapy cycles.

***ESWT was performed as follows:^22^,^23^:*** before the treatment, the pain points of the patients were determined and marked similarly to the method used for the control group. BHSW ballistic shock wave therapy instrument (manufacturer: Weihai Bohua Medical Equipment Co., Ltd.) was used for ESWT. During the treatment, the frequency was set at 8~10Hz and the pressure was 1.5~3.0 bar. During the treatment, the impact dose was selected as 2000 times, and the handle pressure remained medium. The treatment frequency was once every 4~5 days, for a total of four treatment cycles. During the treatment, the intensity, and times of shock wave were adjusted according to patient’s pain feedback to ensure optimal curative effect and safety.

Basic information and relevant indicators were collected by trained nurses for all the patients after completing four cycles of treatment, and included: (1) Pain score of each cycle before and after treatment. The degree of pain was assessed by visual analogue scale (VAS).^24^ The degree of pain was then graded as mild (1~3 points), moderate (4~6 points) or severe (7~10 points). (2) The improvement of limb function before and after four cycles of treatment. Fugl Meyer motor function scale^25^ was used to evaluate the lower limb function of patients, and included 17 items with a maximum score of 34 points. Each item is scored based on a 3-point ordinal scale (0=cannot perform, 1=performs partially and 2=performs fully). A total score of 21 or higher indicated better mobility function.^25^ (3) Serum levels of β-endorphin (β-EP), prostaglandin E2 (PGE2) and nitric oxide (NO) before and after the treatment. Briefly, 5ml of venous blood was extracted under fasting condition, and the relevant indexes were detected in the serum by enzyme-linked immunosorbent assays using respective kits (Shanghai enzyme linked biology) according to manufacturer’s instructions.

### Statistical analysis:

The sample size was calculated using the Sample Size Calculator (http://riskcalc.org:3838/samplesize/) based on 0.9 power at the 5% level of significance.^26^ SPSS 22.0 software was used for data analysis. Descriptive statistics was used to describe the general data of the patients. Shapiro-Wilk test was used to test the normality of the measurement data. The measurement data were presented as mean±standard deviation and compared by using t-test and variance analysis. The counting data are presented as numbers and percentages (n, %) and compared by using *χ^2^* test. The difference was considered statistically significant when P < 0.05.

## RESULTS

A total of 107 patients met the inclusion criteria of this retrospective study. Of them, 51 patients received core muscle training + TMT and 56 received core muscle training + ESWT. There was no significant difference in age, course of disease and other related basic data between the two groups (*P*>0.05) Table-I. Before the treatment, there was no significant difference in the VAS scores between the two groups (*P*>0.05). After the treatment, VAS scores of both groups were significantly lower than those before the treatment. At the same time, VAS scores of each cycle in the observation group were significantly lower than those in the control group (*P*<0.05) Table-II. There was no significant difference in limb function between the two groups before the treatment (*P*>0.05). After four cycles of treatment, the proportion of patients with mild limb dysfunction in the observation group increased significantly, and the proportion of patients with severe and obvious dysfunction decreased significantly. Compared with the control group, the difference was statistically significant (*P*<0.05) Table-III. There was no difference in the levels of β-EP, PGE2 and NO between the two groups before the treatment (*P*>0.05). After the treatment, the levels of β-EP in the observation group were significantly higher than those in the control group, but the levels of PGE2 and NO were significantly lower than those in the control group (*P*<0.05) Table-IV.

**Table-I:** Basic characteristics of the patients [n (%),*χ̅*±*S*]

Group	n	Sex ratio	Age (years)	Pain duration (months)

		^(Male^/Female^)^		
Control group	51	26/25	30~67 (52.62±9.91)	2~27 (12.64±6.17)
Observation group	56	31/25	31~69 (51.37±9.39)	4~25 (11.51±4.84)
*χ^2^*/*t*	-	0.205	0.669	1.058
*P*	-	0.650	0.505	0.293

**Table-II:** Comparison of VAS scores before and after the treatment (Fraction,*χ̅*±*S*)

Group	n	Before therapy	Treatment week 1	Treatment week 2	Treatment weeks 3	Treatment for 1 month
Control group	51	6.72±1.41	6.07±1.05	5.12±0.86	4.29±0.70	3.64±0.59
Observation group	56	7.08±1.21	5.23±1.01	4.08±0.85	3.20±0.72	2.66±0.66
t	-	1.433	4.240	6.335	7.950	8.038
P	-	0.155	<0.001	<0.001	<0.001	<0.001

**Table-III:** Comparison of Fugl-Meyer scale scores before and after treatment [n(%)].

Group	n	Before therapy		After treatment	

		High mobility function (≥21 score)	Low mobility function (<21 score)	High mobility function (≥21 score)	Low mobility function (<21 score)
Control group	51	23(45.1)	28(54.9)	42(82.35)	9(17.64)
Observation group	56	26(46.43)	30(53.57)	53(94.64)	3(5.36)
*χ^2^*	-	2.014		7.904	
P	-	0.569		0.048	

**Table-IV:** Comparison of NO, β-EP and PGE2 levels before and after the treatment (*χ̅*±*S*).

Group	n	NO (pmol/L)		β-EP (ng/L)		PGE2 (pg/mL)	

		Before therapy	After treatment	Before therapy	After treatment	Before therapy	After treatment
Control group	51	5.11±1.26	2.70±1.04	166.03±20.48	211.33±21.87	20.88±3.54	15.49±3.10
Observation group	56	5.48±1.23	1.69±0.76	163.57±19.42	256.23±22.06	21.23±3.94	6.58±2.27
t	-	1.510	5.666	0.640	10.556	0.481	16.780
P	-	0.134	<0.001	0.524	<0.001	0.632	<0.001

## DISCUSSION

This study found that ESWT and TMT were associated with significant pain relief and limb function improvement in patients with LBP. We showed that the therapeutic effect of ESWT was significantly better than that of TMT. Shock wave mainly promotes water explosion through high pressure, and then generates acoustic energy. This acoustic wave is concentrated into high-energy shock wave after being reflected by the reflector that can cause physical shock, stimulate the release of auxin and promote angiogenesis.^27^ Therefore, ESWT may promote tissue repair and pain relief and can potentially be effective in the treatment of chronic pain caused by fracture nonunion and myofascial lesions.^28^

In the study of Zhang L et al.^29^ 30 patients with chronic LBP were treated with ESWT. After four weeks of the treatment, the VAS score, Oswestry low back pain dysfunction index (ODI) and Beck Depression Index (BDI) of the patients in this group were significantly lower than those in the pre-treatment and physiotherapy groups. The studies by Walewicz et al.^16^ and Lee et al.^17^ also reported that ESWT can significantly reduce pain and improve the general functional state. However, a recent study by Rajfur et al.^15^ reported that ESWT has a role in reducing pain, but no significant effect on improving functional state. In our study, our results were consistent with the conclusions made by Zhang, Walewicz and Lee et al.^17^ The mechanism of action of the ESWT involves cell membrane destruction at the pain site as a result of the mechanical stress effect, cavitation effect and osmotic effect of the shock wave. This in turn leads to the release of large amounts of β-EP that can inhibit generation and transmission of pain signals and reduce pain sensitivity of the body. Moreover, extracorporeal shock wave therapy can promote effective muscle relaxation, release the adhesive soft tissue at the pain site, stimulate cell activation, alleviate local inflammatory reaction and prevent infiltration and exudation of inflammatory cells. In addition, shock wave therapy has a good nerve block effect, may improve pain inhibition effect and accelerate soft tissue repair. Xie K et al.^30^ showed that the application of ESWT have a significant impact on pain signal transmission, affects the release of substance P, promotes the effective improvement of local blood circulation, and has an overall analgesic effect. In our study, ESWT was significantly better than TMT in controlling inflammatory response. The reduction of NO and PGE2 levels can effectively activate the analgesic mechanism and relieve the degree of pain.^31^ β-EP is a very important endogenous opioid peptide neurotransmitter in the body. It has strong analgesic effect through the inhibition of the pain conduction pathway.^32^,^33^ The relief of pain ensures patients’ ability and willingness to participate in rehabilitation exercises, improves patients’ sleep quality and psychological state, and promotes better overall recovery of limb function.

In addition, despite the efficacy of ESWT in treating LBP, we need to take into account the high cost of ESWT in clinical practice, especially in low- and middle-income countries. It is known that the shockwave devices may cost over $20,000 to $30,000^34^, and although the cost for shockwave therapy varies from case to case, the average cost of low-energy ESWT is estimated to be around $900 or more, which is significantly higher that other pain management modalities such as massage and TENS.^35^,^36^ Therefore, cost-effectiveness of treatment modalities should also be considered in the decision making of interventions for LBP patients.

### Limitations of the study:

One limitation is that it is a single-center retrospective analysis, with small sample size and small observation indicators which may decrease the validity of our findings.^37^ Another limitation is that our study only relies on the analysis of the data collected after four weeks of treatment. Further multi-center studies with large sample sizes and longer treatment cycle are needed to explore the treatment of LBP. Finally, in our study, the cutoff score of the lower-extremity motor subscale of Fugl-Meyer assessment was based on the study by Kwong et al.^25^, but different cutoff may affect the comparison results of patients mobility function.

## CONCLUSION

ESWT is better than TMT in the treatment of pain in patients with LBP. The results of this study can provide reference for medical practitioners.

### Authors’ contributions:

**TW** conceived and designed the study.

**DW, XZ, JL and BY** collected the data and performed the analysis.

**TW** was involved in the writing of the manuscript, is responsible for the integrity of the study.

All authors have read and approved the final manuscript.
